# Enhanced Detection Precision of the Taiji Program by Frequency Setting Strategy Based on a Hierarchical Optimization Algorithm

**DOI:** 10.3390/s23239431

**Published:** 2023-11-27

**Authors:** Jiafeng Zhang, Zhen Yang, Xiaoshan Ma, Xiaodong Peng, Chen Gao, Mengyuan Zhao, Wenlin Tang

**Affiliations:** 1 Key Laboratory of Electronics and Information Technology for Space System, National Space Science Center, Chinese Academy of Sciences, Beijing 100190, China; 2 University of Chinese Academy of Sciences, Beijing 100049, China; 3 Institute of Engineering Thermophysics, Chinese Academy of Sciences, Beijing 100190, China; 4 School of Fundamental Physics and Mathematical Sciences, Hangzhou Institute for Advanced Study, UCAS, Hangzhou 310024, China; 5 Taiji Laboratory for Gravitational Wave Universe, Hangzhou 310024, China; 6 Key Laboratory of Gravitational Wave Precision Measurement of Zhejiang Province, Hangzhou 310024, China

**Keywords:** frequency distribution scheme, Taiji program, hierarchical optimization algorithm, laser interferometry system

## Abstract

For space-based gravitational wave detection, a laser interferometric measurement system composed of a three-spacecraft formation offers the most rewarding bandwidth of astrophysical sources. There are no oscillators available that are stable enough so that each spacecraft could use its own reference frequency. The conversion between reference frequencies and their distribution between all spacecrafts for the synchronization of the different metrology systems is the job of the inter-spacecraft frequency setting strategy, which is important for continuously acquiring scientific data and suppressing measurement noise. We propose a hierarchical optimization algorithm to solve the frequency setting strategy. The optimization objectives are minimum total readout displacement noise and maximum beat-note frequency feasible range. Multiple feasible parameter combinations were obtained for the Taiji program. These optimized parameters include lower and upper bounds of the beat note, sampling frequency, pilot tone signal frequency, ultrastable clock frequencies, and modulation depth. Among the 20 Pareto optimal solutions, the minimum total readout displacement noise was 4.12 $\mathrm{pm}/\sqrt{\mathrm{Hz}}$, and the maximum feasible beat-note frequency range was 23 MHz. By adjusting the upper bound of beat-note frequency and laser power transmitted by the telescope, we explored the effects of these parameters on the minimum total readout displacement noise and optimal local laser power in greater depth. Our results may serve as a reference for the optimal design of laser interferometry system instrument parameters and may ultimately improve the detection performance and continuous detection time of the Taiji program.

## 1. Introduction

In 2016, the Laser Interferometer Gravitational-Wave Observatory (LIGO) [1] successfully detected gravitational wave phenomena that proved the existence of gravitational waves. Space-based gravitational wave detection missions have been proposed and performed in recent years because of the surface vibrations of the Earth, the noise of the gravitational gradient, and limitations of the ground test baseline length [2]. These missions include the Laser Interferometer Space Antenna (LISA) mission [3,4] and the Taiji [5,6,7] and Tianqin programs [8]. Space-based gravitational-wave detection usually uses the laser interferometry principle [9] and employs a three-spacecraft constellation placed in an equilateral triangle [3,4,5,6,8]; many functions are achieved by laser beams exchanged between the spacecrafts (S/Cs), such as scientific interferometry, absolute inter-spacecraft distance measurements, digital data communication, and clock-noise transfer [10,11,12].

In a three-spacecraft constellation, the laser on one S/C interferes with the laser received from another S/C. The beat-note signal of two laser is digitized by an analog-to-digital converter (ADC) and analyzed by a high-precision phasemeter (PM). Both the ADC and the PM are triggered by an ultrastable oscillator (USO) that provides a time reference. The frequency instability of the trigger signal introduces additional ranging noise, affecting the arm length measurements. Additionally, the inherent jitter of the ADC distorts the sampling process. A pilot tone (i.e., a stable sinusoidal reference signal derived from the USO) has been inserted to correct the clock and ADC noise [6,13,14]. Considering the differential jitter and relative drift of three onboard USOs on different S/Cs, a clock-tone transfer chain has been proposed via an electro-optic modulator (EOM) to modulate the USO signal to sidebands on the outgoing beam to remove clock noise and correct the relative clock drift by using postprocessing [10,11,12]. To realize the functions listed above, the frequencies of the USO, sideband, pilot tone, ADC sampling, and beat note must be comprehensively considered and optimized. Moreover, total laser power is a limited resource used to ensure the effectiveness of scientific signal interference, and the readout displacement noise of the signal is affected by the power ratio of the sideband and carrier. The power ratio needs to be reasonably designed to minimize readout displacement noise.

Different frequency-setting schemes have been established for the LISA mission to address different concerns. For example, Kullmann [15] conducted a detailed, in-depth device experiment for the setting of the ADC sampling and pilot-tone frequencies involved in the LISA mission. They concluded that when the pilot-tone frequency was 72 MHz and the ADC sampling frequency was 50 MHz, sampling the beat-note frequency from 2 to 20 MHz could meet the noise requirements of this component of LISA. Barke [13] designed an inter-satellite frequency distribution scheme for the LISA program for beat-note frequencies in the range of [7 MHz, 23 MHz]; the ADC sampling and pilot-tone frequencies were 80 and 75 MHz, respectively. Zhang [16] analyzed the Taiji mission orbit and a possible phase-locking scheme and developed an offset frequency-setting scheme for beat-note frequencies in the range of [5 MHz, 25 MHz]. Although all these schemes have considered the problem of frequency setting in space-based gravitational-wave detection missions from different perspectives, they have not provided complete constraints or optimal setting schemes that include the beat-note frequency range as well as the frequencies of USO, ADC sampling, pilot tone, and sideband, and none of them consider the coupling relationship between frequency parameters. The unified consideration of these factors avoids the one-sidedness of individual parameter settings and reduces unnecessary redundancy between parameters, further improving the detection capability.

In this study, a frequency distribution scheme using a hierarchical optimization algorithm is introduced for the Taiji program by considering the frequency bands or frequencies for each function or device to ensure an accurate readout of the inter-spacecraft heterodyne signal, synchronize the onboard clock with those on other S/Cs, and generate an ADC sampling frequency and pilot tone signal.

The remainder of this paper is organized as follows. Section 2 introduces frequency factors and constraints, such as sideband frequency, ADC sampling, and pilot-tone frequencies. The optimization algorithm is introduced in Section 3. Section 4 describes the solution for the frequency setting scheme for each function or device. The effects of key parameters and total readout displacement noise are also analyzed. Finally, Section 5 concludes this study.

## 2. Frequency Factors and Constraints

In space-borne, gravitational-wave detection missions, laser links need to accomplish ultralong-range laser interference and various auxiliary functions, such as clock-noise transfer, pseudorandom noise (PRN) ranging, and information transfer [10,17]. The Doppler shift affects the beat-note signal generated by heterodyne interference; therefore, offset frequencies need to be added to the phase-locking process to make them fall into a reasonable range. The signal is sampled and analyzed by the ADC and PM and triggered by the USO onboard. The USO signal is first multiplied to the GHz level and imprinted on the sidebands of the outgoing laser beam by EOM to perform inter-spacecraft clock transfer. Setting the sideband frequency and power ratio will affect the size of the readout displacement noise. The USO is then multiplied to the MHz level to provide the internal clock signal for the ADC and PM operations and construct the pilot tone to eliminate ADC sampling jitter and clock noise. In this process, some parameters have complex mutual constraints and need to be optimized systematically.

### 2.1. Readout Displacement Noise and Sideband Frequency Constraints

**Figure 1:**
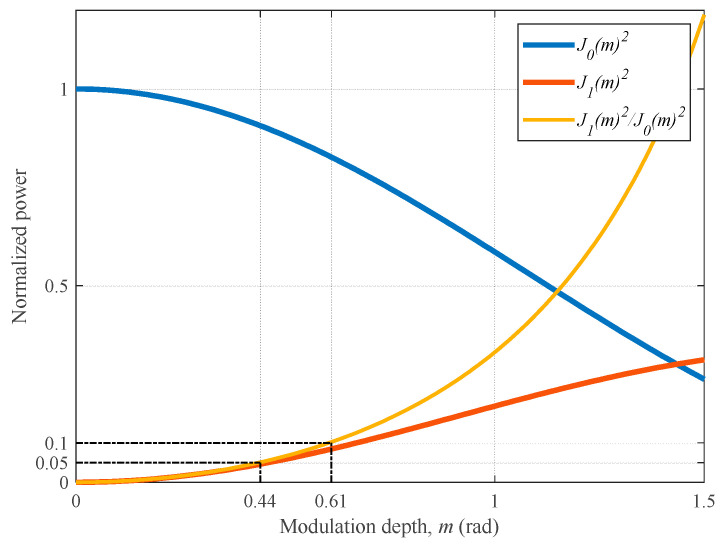
Carrier, first-order sideband (normalized power over the modulation depth *m*), and the ratio between the first-order sideband and carrier.

The Taiji program requires that the total readout displacement noise be as small as possible, which includes carrier and first-order sideband readout displacement noise. Moreover, based on the experience of the LISA mission [13,14,18], the first-order sideband readout displacement noise cannot be higher than 1/10 of the total readout displacement noise. The power ratio between the first-order sidebands and carrier could be expressed by the ratio of the squares of first- and zero-orders of the Bessel functions of the first kind:

(1) $$\frac{P_{\mathrm{sideband}}}{P_{\mathrm{carrier}}}=\frac{J_{1}{\left(m\right)}^{2}}{J_{0}{\left(m\right)}^{2}},$$

where *J*_0_(*m*) and *J*_1_(*m*) denote the zero- and first-orders of the Bessel functions of the first kind, respectively, and *m* is the modulation depth. Research on the LISA mission [14,18] indicates that this power ratio should be in the range of 5% to 10%, and that the corresponding modulation depth *m* is in the range of [0.44, 0.61], as shown in Figure 1.

Therefore, the optimization objective and constraints are

(2) $$\begin{array}{l} Min\left(\delta x^{\mathrm{total}}\right)\\ \left\{ \begin{array}{l} \delta x^{\mathrm{sideband}}<\frac{1}{10}\delta x^{\mathrm{total}}\\ 0.44<m<0.61 \end{array}\right. \end{array},$$

where $\delta x^{\mathrm{sideband}}$ and $\delta x^{\mathrm{total}}$ indicate the sideband and total readout displacement noise, respectively. The expressions of the readout displacement noise of the carrier and first-order sideband and the total readout displacement noise are [13]

(3) $$\left\{ \begin{array}{l} \delta x_{}^{\mathrm{carrier}}=\frac{\lambda }{2\pi }\frac{1}{J_{0}{\left(m\right)}^{2}}\delta \phi_{\mathrm{total}}\\ \delta x_{}^{\mathrm{sideband}}=\frac{1}{\sqrt{2}}\frac{\lambda }{2\pi }\frac{f_{\mathrm{het}}}{f_{\mathrm{USO}}}\frac{1}{J_{1}{\left(m\right)}^{2}}\delta \phi_{\mathrm{total}}\\ \delta x_{}^{\mathrm{total}}=\delta x_{}^{\mathrm{sideband}}+\delta x_{}^{\mathrm{carrier}} \end{array}\right.,$$

respectively, where λ is the laser wavelength, which is 1064 nm in the Taiji program; *f*_het_ is the beat-note frequency; and *f*_USO_ is the USO frequency. Given that two identical sidebands are generated on the left- and right-hand sides of the carrier after modulation by EOM, a factor of $1/\sqrt{2}$ must be added to the calculation of the sideband frequency noise. $\delta \phi_{\mathrm{total}}$ is the total readout phase noise and contains three components, which is shown as follows [13,19]:

(4) $$\left\{ \begin{array}{l} \delta \phi_{\mathrm{total}}=\sqrt{\delta \phi_{\mathrm{SN}}^{2}+\delta \phi_{\mathrm{RIN}}^{2}+\delta \phi_{\mathrm{EN}}^{2}}\\ \delta \phi_{\mathrm{SN}}=\sqrt{\frac{2e(P_{\mathrm{local}}+P_{\mathrm{receive}})}{R_{\mathrm{pd}}\varepsilon_{\mathrm{het}}P_{\mathrm{local}}P_{\mathrm{receive}}}}\\ \delta \phi_{\mathrm{RIN}}=\mathrm{RIN}\sqrt{\frac{P_{\mathrm{local}}^{2}+P_{\mathrm{receive}}^{2}}{2\varepsilon_{\mathrm{het}}P_{\mathrm{local}}P_{\mathrm{receive}}}}\\ \delta \phi_{\mathrm{EN}}=\frac{\sqrt{2N_{\mathrm{pd}}}}{R_{\mathrm{pd}}}\sqrt{\frac{\delta I_{\mathrm{pd}}^{2}+2\pi C_{\mathrm{pd}}f_{\mathrm{upper}}\delta U_{\mathrm{pd}}}{\varepsilon_{\mathrm{het}}P_{\mathrm{local}}P_{\mathrm{receive}}}} \end{array}\right.,$$

where $\delta \phi_{\mathrm{SN}}$, $\delta \phi_{\mathrm{RIN}}$, and $\delta \phi_{\mathrm{EN}}$ are the shot, RIN, and electronic noise, respectively; and *P*_local_ and *P*_receive_ represent the local laser power and laser power received by the telescope, respectively. *P*_receive_ [12] is calculated as

(5) $$P_{\mathrm{receive}}=0.4073\times \frac{\pi^{2}D^{4}P_{\mathrm{tel}}\varepsilon_{\mathrm{opt}}}{8L^{2}\lambda^{2}}.$$

For the Taiji program, the explanation and values of other parameters in Equations (4) and (5) are listed in Table 1.

In Equation (3), $\delta x_{}^{\mathrm{sideband}}$ is positively correlated with $f_{\mathrm{het}}$ and negatively correlated with $f_{\mathrm{USO}}$. Because the beat-note frequency signal $f_{\mathrm{het}}$ varies with time, $\delta x_{}^{\mathrm{sideband}}$ is usually calculated by replacing *f*_het_ with *f*_upper_. From Equations (3) and (4), the total readout displacement noise may be minimized by reasonably setting *f*_upper_, *f*_USO_, *m*, *P*_receive_, and *P*_local_.

### 2.2. Constraints of the ADC Sampling and Pilot-Tone Frequencies with the Beat-Note Frequency

According to Nyquist’s theorem, the sampling frequency of the ADC needs to be greater than at least two times the beat-note frequency. That is,

(6) $$f_{\mathrm{ADC}}>2f_{\mathrm{upper}}.$$

The USO is used to control the ADC sampling frequency and construct the pilot-tone signal; the divider or synthesizer can realize this process. According to Heinzel [10], the noise introduced by the dividers is much smaller than that introduced by the synthesizers, and, therefore, the frequency division approach is typically used. In the actual application process, integer frequency division is usually chosen.

The aliasing signal generated by the ADC sampling of the pilot-tone signal interferes with the beat-note frequency measurement. For example, when the sampling frequency of the ADC is 82 MHz and the frequency of the pilot-tone signal is 80 MHz, the frequency of the aliased signal generated by the ADC in the under-sampled pilot-tone signal will be 2 MHz. Therefore, the frequency of the aliased signal must not overlap the beat-note frequency range. This constraint can be expressed by the following equation:

(7) $$|f_{\mathrm{ADC}}-f_{\mathrm{PT}}|<f_{\mathrm{lower}}.$$

Therefore, setting the pilot-tone signal frequency *f*_PT_ and ADC sampling frequency *f*_ADC_ imposes the following constraints:

(8) $$\left\{ \begin{array}{l} n_{1}*f_{\mathrm{ADC}}=f_{\mathrm{USO}}\\ n_{2}*f_{\mathrm{PT}}=f_{\mathrm{USO}}\\ f_{\mathrm{ADC}}>2f_{\mathrm{upper}}\\ \left|f_{\mathrm{ADC}}-f_{\mathrm{PT}}\right|<f_{\mathrm{lower}}\\ f_{\mathrm{USO}}<5\;\mathrm{GHz}\\ f_{\mathrm{PT}}<98\;\mathrm{MHz} \end{array}\right.\;\;\;\;\;n_{1},n_{2}\;are\;integers,$$

where the value of 5 GHz is the artificial upper bound set for *f*_USO_ to constrain it to finite values. Based on Kullmann’s [15] study, the upper bound of *f*_PT_ is set to 98 MHz to avoid poor performance of the pilot tone correction.

## 3. Hierarchical Optimization Algorithm

In this section, the optimization model is introduced in Section 3.1, and then the hierarchical optimization algorithm for this optimization model is introduced in Section 3.2.

### 3.1. Optimization Model

According to the analysis in Section 2, the frequency of the mission operation involves four terms: the beat-note *f*_het_, the USO *f*_USO_, the pilot-tone signal *f*_PT_, and the ADC sampling *f*_ADC_ frequencies. The variable *f*_het_ is affected by the Doppler shift and changes dynamically over time, while the remaining three terms remain unchanged. The variation range of the inter-satellite beat-note frequency *f*_het_ is determined by the lower and upper bounds of the beat-note frequency, namely *f*_lower,_ and *f*_upper_. Therefore, the optimization goal is to make the sideband readout displacement noise meet the mission requirement, minimize the total readout displacement noise, and maximize the feasible range of beat-note frequency by reasonably allocating the values of each frequency or frequency band, and modulation depth *m*, respectively. The optimization model is as follows:

(9) $$\begin{array}{l} \Gamma_{1}(f_{\mathrm{upper}},f_{\mathrm{USO}},f_{\mathrm{PT}},f_{\mathrm{ADC}},m,P_{\mathrm{local}})=\min\left(\delta x^{\mathrm{total}}\right)\\ \Gamma_{2}(f_{\mathrm{upper}},f_{\mathrm{lower}})\;\;\;\;\;\;\;\;\;\;\;\;\;\;\;\;\;\;\;\;\;\;\;\;\;\;\;\;\;\;=\max\left(f_{\mathrm{upper}}-f_{\mathrm{lower}}\right)\\ s.t.\\ \left\{ \begin{array}{l} \delta x^{\mathrm{sideband}}<\frac{1}{10}\delta x^{\mathrm{total}}\\ n_{1}*f_{\mathrm{ADC}}=f_{\mathrm{USO}}\\ n_{2}*f_{\mathrm{PT}}=f_{\mathrm{USO}}\\ f_{\mathrm{ADC}}>2f_{\mathrm{upper}}\\ \left|f_{\mathrm{ADC}}-f_{\mathrm{PT}}\right|<f_{\mathrm{lower}}\\ f_{\mathrm{lower}}>=2\;\mathrm{MHz}\\ f_{\mathrm{upper}}<=25\;\mathrm{MHz}\\ f_{\mathrm{USO}}<5\;\mathrm{GHz}\\ f_{\mathrm{PT}}<98\;\mathrm{MHz}\\ 0.44<m<0.61 \end{array}\right.\;\;\;\;\;n_{1},n_{2}\;are\;integers, \end{array}$$

where $\Gamma_{1}$ and $\Gamma_{2}$ represent the two objective functions.

### 3.2. Optimization Process

Owing to the multiple objectives and parameters involved in the optimization solution, a hierarchical optimization approach is used for this optimization model based on computational efficiency considerations when selecting *f*_USO_, *f*_PT_, *f*_ADC_, *f*_upper_, *f*_lower_, and *m*. The optimization process is as follows.

Step 1: A multi-objective optimization algorithm is used to set the lower and upper bounds of the beat-note frequency (*f*_lower_, *f*_upper_), USO frequency *f*_USO_, and sideband modulation depth *m*. The multi-objective optimization model for this step is expressed by Equation (10).

(10) $$\begin{array}{l} \left\{ \begin{array}{l} \Gamma_{1}(f_{\mathrm{upper}},f_{\mathrm{USO}},\bar{f_{\mathrm{PT}}},\bar{f_{\mathrm{ADC}}},m,\bar{P_{\mathrm{local}}})=\min\left(\delta x^{\mathrm{total}}\right)\\ \Gamma_{2}(f_{\mathrm{lower}})\;\;\;\;\;\;\;\;\;\;\;\;\;\;\;\;\;\;\;\;\;\;\;\;\;\;\;\;\;\;\;\;\;\;\;\;\;\;\;\;=\max(f_{\mathrm{lower}})\\ \Gamma_{3}(f_{\mathrm{upper}})\;\;\;\;\;\;\;\;\;\;\;\;\;\;\;\;\;\;\;\;\;\;\;\;\;\;\;\;\;\;\;\;\;\;\;\;\;\;\;=\min(f_{\mathrm{upper}})\\ \Gamma_{4}(f_{\mathrm{upper}},f_{\mathrm{lower}})\;\;\;\;\;\;\;\;\;\;\;\;\;\;\;\;\;\;\;\;\;\;\;\;\;\;\;\;\;=\max(f_{\mathrm{upper}}-f_{\mathrm{lower}}) \end{array}\right.\\ s.t.\\ \left\{ \begin{array}{l} 0.44<m<0.61\\ f_{\mathrm{USO}}<5\;\mathrm{GHz}\\ 2\;\mathrm{MHz}<f_{\mathrm{lower}}<10\;\mathrm{MHz}\\ 20\;\mathrm{MHz}<f_{\mathrm{upper}}<=25\;\mathrm{MHz} \end{array}\right. \end{array}.$$

In Equation (10), $\Gamma_{x}$ denotes the x-th optimization objective. In $\Gamma_{1}$, where $\bar{f_{\mathrm{PT}}}$, $\bar{f_{\mathrm{ADC}}}$ and $\bar{P_{\mathrm{local}}}$ denote that the variables are taken as constant. $\Gamma_{2}$ and $\Gamma_{3}$ in Equation (10) are the two objective functions derived from $\Gamma_{1}$ in Equation (9) according to Equations (3) and (8), respectively. $\Gamma_{2}$ aims to expand the selection spaces of *f*_ADC_ and *f*_PT_, and $\Gamma_{3}$ aims to reduce the sideband readout displacement noise, which is proportional to *f*_upper_. The objective function $\Gamma_{4}$ aims to maximize the feasible range of the beat-note frequency, which is the opposite of $\Gamma_{2}$ and $\Gamma_{3}$. In actual mission operations, *f*_upper_, *f*_lower_, and *f*_USO_ are commonly set as integers. To reduce the complexity of the optimization problem while determining the solution, *f*_upper_, *f*_lower_, and *f*_USO_ are not constrained as integers in this step.

In the current Taiji program, the upper bound of the beat-note frequency is 25 MHz. Using the parameter values listed in Table 1, P_receive_ = 2.154 × 10^−9^ W. Therefore, the values of all parameters in Equation (4), except P_local_, are obtained. Because P_local_ appears in both the numerator and denominator, the optimal value of P_local_, which is 2.06 × 10^−3^ W in the current parameter settings, can be derived by simply minimizing. When calculating $\delta x^{\mathrm{total}}$ in this step, the values of *P*_local_ = 2.06 × 10^−3^ W and the parameters in Table 1 are used by default.

Step 2: The values of *f*_lower_, *f*_upper_, and *f*_USO_ obtained in Step 1 are adjusted to be integers:

(11) $$\left\{ \begin{array}{l} f_{\mathrm{lower}}^{\mathrm{new}}=\mathrm{floor}(f_{\mathrm{lower}})\\ f_{\mathrm{upper}}^{\mathrm{new}}=\mathrm{floor}(f_{\mathrm{upper}})\\ f_{\mathrm{USO}}^{\mathrm{new}}=\mathrm{floor}(f_{\mathrm{USO}}) \end{array}\right.,$$

where $f_{\mathrm{lower}}^{\mathrm{new}}$, $f_{\mathrm{upper}}^{\mathrm{new}}$, and $f_{\mathrm{USO}}^{\mathrm{new}}$ represent the adjusted values of *f*_lower_, *f*_upper_, and *f*_USO_, respectively.

Step 3: Exhaustive enumeration is used to search for possible $\left[f_{\mathrm{USO}}^{},f_{\mathrm{PT}},f_{\mathrm{ADC}}\right]$ combinations. The values of *f*_PT_ and *f*_ADC_ need to satisfy the following constraints:

(12) $$\left\{ \begin{array}{l} f_{\mathrm{ADC}}>=2f_{\mathrm{upper}}^{\mathrm{new}}\\ 1<|f_{\mathrm{ADC}}-f_{\mathrm{PT}}|<f_{\mathrm{lower}}^{\mathrm{new}} \end{array}\right..$$

Suppose there are *n* possible combinations stored in the following matrix:

(13) $$\left[\begin{array}{l} f_{\mathrm{USO}}^{1},f_{\mathrm{PT}}^{1},f_{\mathrm{ADC}}^{1}\\ f_{\mathrm{USO}}^{2},f_{\mathrm{PT}}^{2},f_{\mathrm{ADC}}^{2}\\ \ldots \\ f_{\mathrm{USO}}^{n},f_{\mathrm{PT}}^{n},f_{\mathrm{ADC}}^{n} \end{array}\right]$$

The combination such that $f_{\mathrm{USO}}^{j},j=1,2,\ldots ,n$ is closest to $f_{\mathrm{USO}}^{\mathrm{new}}$ is chosen. If $f_{\mathrm{USO}}^{m}$ is closest to $f_{\mathrm{USO}}^{\mathrm{new}}$, then $f_{\mathrm{USO}}^{\mathrm{new}}=f_{\mathrm{USO}}^{m}$ is updated. To distinguish this value from $f_{\mathrm{USO}}^{\mathrm{new}}$ obtained in Step 2, the updated $f_{\mathrm{USO}}^{\mathrm{new}}$ obtained in this step is referred to as $f_{\mathrm{USO}}^{\mathrm{opt}}$.

Step 4: *P*_local_ is updated according to $f_{\mathrm{upper}}^{\mathrm{new}}$, and then the modulation depth *m* is updated according to $\left[f_{\mathrm{upper}}^{\mathrm{new}},f_{\mathrm{USO}}^{\mathrm{opt}}\right]$ from Steps 2 and 4. The updating method is as follows:

(14) $$\begin{array}{l} \min\left(\delta \phi_{\mathrm{SN}}^{2}+\delta \phi_{\mathrm{RIN}}^{2}+\delta \phi_{\mathrm{EN}}^{2}\right)\\ \left\{ \begin{array}{l} f_{\mathrm{upper}}=f_{\mathrm{upper}}^{\mathrm{new}}\\ P_{\mathrm{receive}}=2.154\times 10^{-9}\;W \end{array}\right.\\ \min\left(\delta x^{\mathrm{total}}(f_{\mathrm{USO}}^{\mathrm{opt}},f_{\mathrm{upper}}^{\mathrm{new}},m)\right)\\ \left\{ \begin{array}{l} \delta x^{\mathrm{total}}(f_{\mathrm{USO}}^{\mathrm{opt}},f_{\mathrm{upper}}^{\mathrm{new}},m)=\delta x^{\mathrm{total}}\left(\frac{f_{\mathrm{upper}}^{\mathrm{new}}}{f_{\mathrm{USO}}^{\mathrm{opt}}}\frac{1}{J_{1}{\left(m\right)}^{2}}+\frac{1}{J_{0}{\left(m\right)}^{2}}\right)\\ m\in [0.44,0.61] \end{array}\right. \end{array}.$$

The minimum value of the total readout displacement noise after updating $\left[f_{\mathrm{upper}}^{\mathrm{new}},f_{\mathrm{USO}}^{\mathrm{opt}}\right]$ is obtained by adjusting the parameter m in the range [0.44, 0.61].

Step 5: Backtracking mechanism. Since the default *P*_local_ value is obtained by assuming *f*_upper_ = 25 MHz, it may change after completing Steps 1–4. Therefore, if *P*_local_ changed, then update *P*_local_ and return to Step 1 until no new *P*_local_ appears.

**Figure 2:**
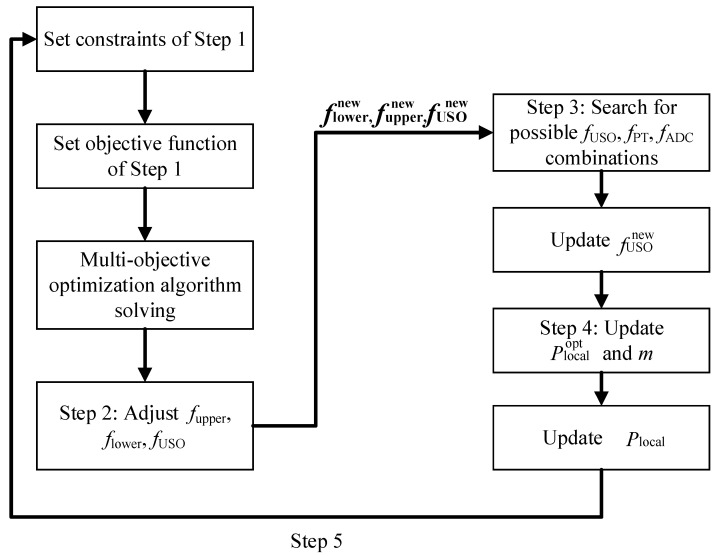
Flowchart of the algorithm.

The flowchart of the algorithm as shown in Figure 2.

## 4. Results and Discussion

### 4.1. Optimization Results

The mathematical model presented in Section 3 was solved using an AMD Ryzen 9 3900X 12-core processor. The time consumption of each step is listed in Table 2. To reduce the complexity associated with the solution of multi-objective problems and improve the convergence efficiency of the solution set, we used one of the most popular multi-objective optimization algorithms: the nondominated sorting genetic algorithm II (NSGA-II) [24].

As a characteristic of multi-objective optimization algorithms, an optimal solution set is usually obtained instead of a single optimal solution to balance the degree of optimization of each objective.

**Figure 3:**
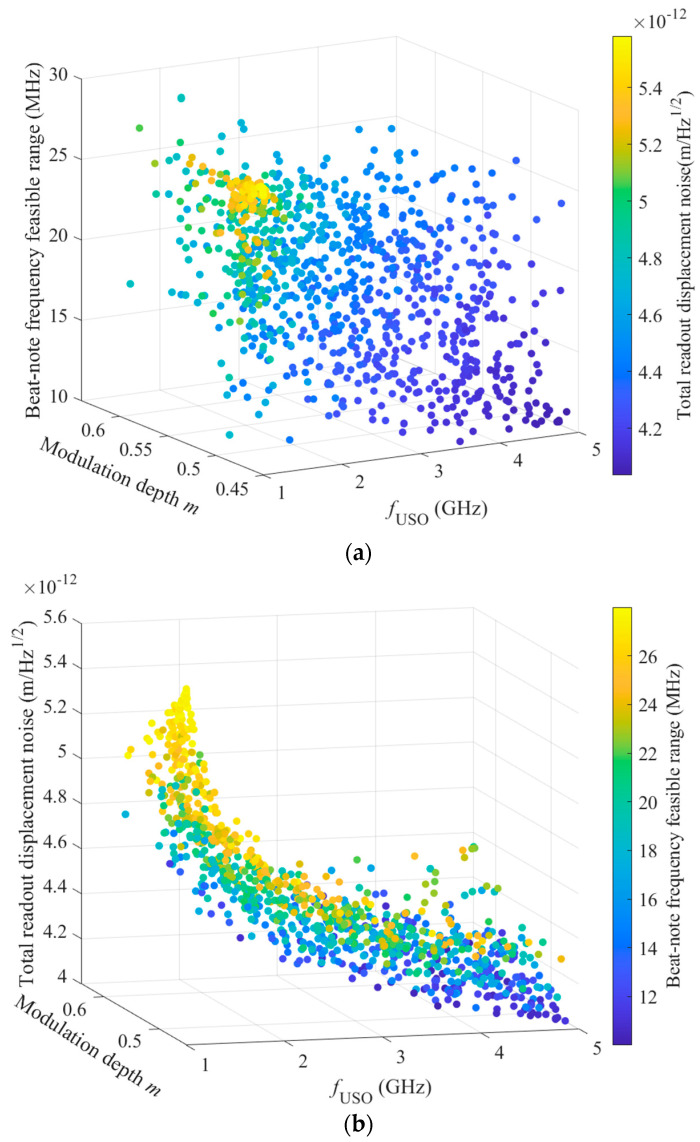
Pareto-optimal solution plane: (**a**) Scatter plot of the total readout displacement noise with the modulation depth; USO frequency; and beat-note frequency in a feasible range along the x-, y-, and z-axes, respectively. The total readout displacement noise $\delta x^{\mathrm{total}}$ is represented by the colored bar. (**b**) Scatter plot of the beat-note frequency in a feasible range with the modulation depth; USO frequency; and total readout displacement noise along the x-, y-, and z-axes, respectively. The beat-note frequency in a feasible range is represented by the colored bar.

The Pareto-optimal solution plane obtained in Step 1 of Section 3.2 is shown in Figure 3.

During the operation of a mission, a smaller total readout displacement noise $\delta x^{\mathrm{total}}$ indicates a better detection of signals, and a larger feasible range of the beat-note frequency, which exists between *f*_upper_ and *f*_lower_, indicates a better offset frequency setting. In Figure 3a, the warmer the color of the scatter plot, the higher the value of $\delta x^{\mathrm{total}}$. Hence, the smaller the value of the beat-note frequency range, the smaller the value of the modulation depth; a larger value of *f*_USO_ corresponds to a smaller value of $\delta x^{\mathrm{total}}$. In Figure 3b, a warmer scatter color indicates a larger beat-note frequency interval; a larger beat-note frequency range corresponds to a larger $\delta x^{\mathrm{total}}$ value. Based on Figure 3, we can conclude that the value of the beat-note frequency range is inversely related to the magnitude of $\delta x^{\mathrm{total}}$. The two targets need to be reasonably balanced via optimization to increase the maximum feasible range of the beat-note frequency and decrease $\delta x^{\mathrm{total}}$.

This planning problem was solved based on the optimization algorithm introduced in Section 3.2. The results of the 20 Pareto-optimal (feasible) solutions obtained from the final solution are listed in Table 3. The units of *f*_lower_, *f*_upper_, *f*_ADC_, *f*_PT_, and *f*_USO_ are MHz, and the total readout displacement noise $\delta x^{\mathrm{total}}$ has units of $\mathrm{pm}/\sqrt{\mathrm{Hz}}$. It is worth noting that these are the optimal 20 solutions by balancing the two optimization objectives.

The results in Table 3 show that, after utilizing the proposed algorithm, the maximum beat-note frequency feasible range is 23 MHz and yields a total readout displacement noise of 4.21 $\mathrm{pm}/\sqrt{\mathrm{Hz}}$, which corresponds to the parameter combination (*f*_lower_, *f*_upper_, *f*_ADC_, *f*_PT_, *f*_USO_, *m*) = (3 MHz, 25 MHz, 92 MHz, 90 MHz, 4140 MHz, 0.44). Additionally, the smallest total readout displacement noise was 4.12 $\mathrm{pm}/\sqrt{\mathrm{Hz}}$, which corresponds to (*f*_lower_, *f*_upper_, *f*_ADC_, *f*_PT_, *f*_USO_, *m*) = (3 MHz, 23 MHz, 96 MHz, 94 MHz, 4512 MHz, 0.44). From Equation (4), different *f*_upper_ values correspond to different optimal *P*_local_ values. Therefore, when *f*_upper_ = 23 MHz, *P*_local_ = 1.92 mW, and *f*_upper_ = 24 MHz, we obtain *P*_local_ = 1.99 mW, *f*_upper_ = 25 MHz, and *P*_local_ = 2.06 mW.

**Figure 4:**
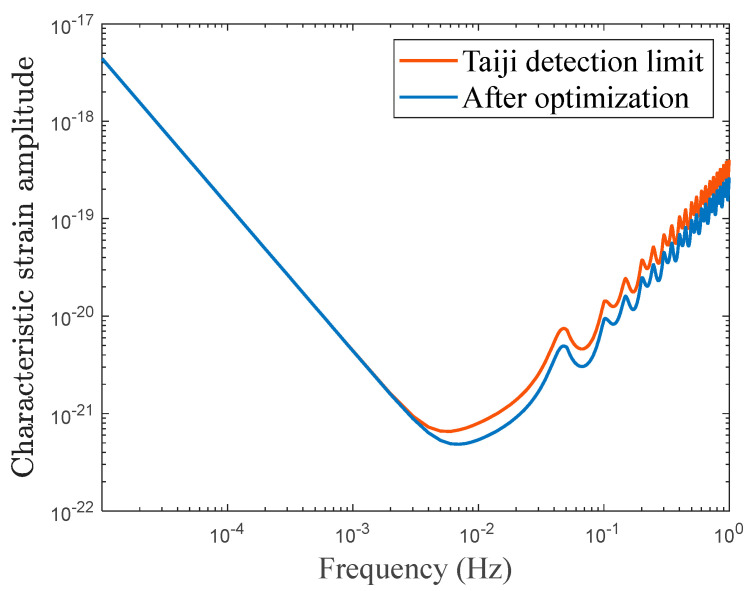
The sensitivity curve of the Taiji program detection limit (red) and after optimization (blue).

According to Taiji mission budget, the position noise is 8 $\mathrm{pm}/\sqrt{\mathrm{Hz}}$ [6], which includes laser frequency noise, readout displacement noise, laser pointing noise, tilt-to-length noise, and so on. Among them, the frequency stability is 30 $\mathrm{Hz}/\sqrt{\mathrm{Hz}}$, the limit of is laser-pointing noise and tile-to-length noise is 1 $\mathrm{pm}/\sqrt{\mathrm{Hz}}$ [25], and the readout displacement noise is about 7.5 $\mathrm{pm}/\sqrt{\mathrm{Hz}}$ [26]. After parameter optimization, the total readout displacement noise is reduced to 4.12 $\mathrm{pm}/\sqrt{\mathrm{Hz}}$. The sensitivity curve of Taiji program detection limit and after optimization with other noise budgets the same, is shown Figure 4. It can be seen from the figure that the optimized parameters have improved the sensitivity in the range of 10 mHz–1 Hz.

### 4.2. Experimental Adjustment of P_tel_ and f_upper_

**Figure 5:**
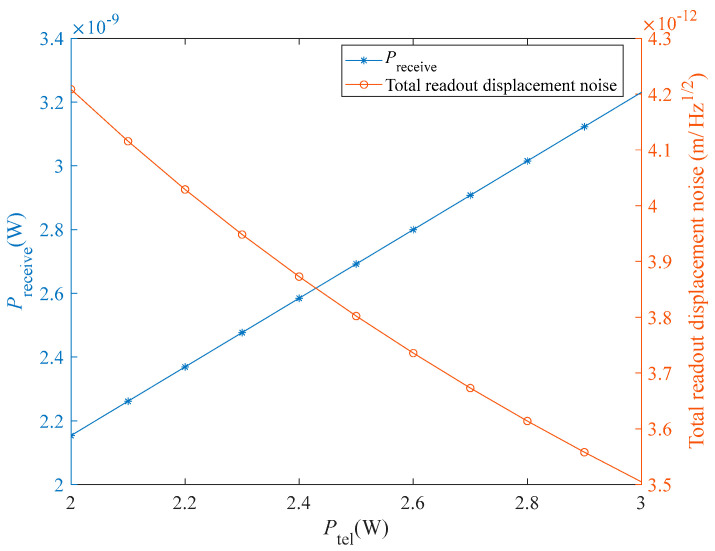
Variation of *P*_receive_ and the total readout displacement noise as a function of *P*_tel_. The x-, left-hand y-, and right-hand y-axes show *P*_tel_, *P*_receive_, and the total readout displacement noise, respectively.

**Figure 6:**
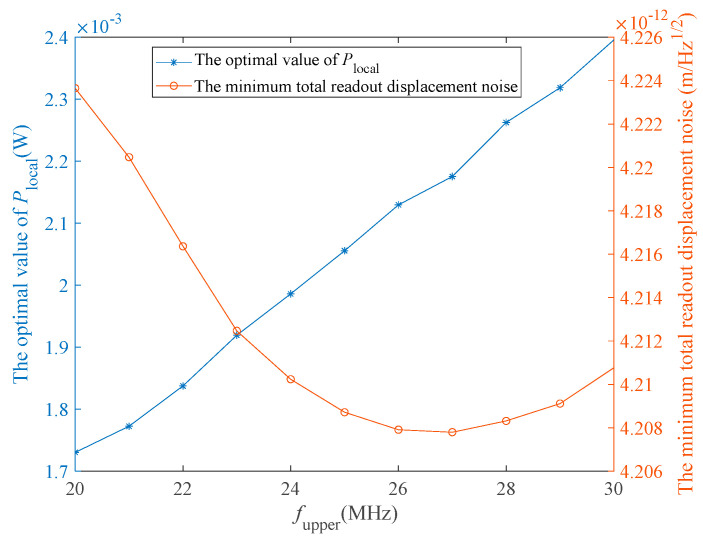
Variations of the optimal *P*_local_ value and the minimum total readout displacement noise with *f*_upper_. The x-, left-hand y-, and right-hand y-axes show *f*_upper_, the optimal *P*_local_, and the minimum total readout displacement noise, respectively.

According to Equation (5), the value of *P*_receive_ is positively proportional to *P*_tel_. The optimal value of *P*_local_ is directly influenced by *f*_upper_. To describe the effect of the values of *P*_tel_ and *f*_upper_ on *P*_receive_ and *P*_local_ in a more intuitive manner, we conducted the following experiments: (1) vary the value of *P*_tel_ in the range [2 W, 3 W] with an interval of 0.1 W and observe the variations of *P*_receive_ and total readout displacement noise; and (2) vary the value of *f*_upper_ in the range [20 MHz, 30 MHz] with an interval of 1 MHz and observe the variations of the optimal *P*_local_ value and minimum total readout displacement noise $\delta x^{\mathrm{total}}$. The results are shown in Figure 5 and Figure 6. It should be noted that in the first experiment, other parameters such as *P*_local_, *m*, and *f*_upper_ were set according to the first optimization results shown in Table 3, while in the second experiment, we set *P*_tel_ = 2 W.

In Figure 5, as *P*_tel_ increases, *P*_receive_ increases linearly, and $\delta x^{\mathrm{total}}$ decreases approximately linearly. In Equation (4) and Step 5 of the optimization algorithm proposed in this study, the variation of *P*_receive_ may cause a change in the optimal *P*_local_ value. However, in practice, the variation of *P*_receive_ is so small that it barely affects the optimal value of *P*_local_. Figure 6 shows the variations of *P*_local_ and $\delta x^{\mathrm{total}}$ with *f*_upper_. The optimal value of *P*_local_ increases as *f*_upper_ increases, while $\delta x^{\mathrm{total}}$ first decreases and then increases. When *f*_upper_ = 27 MHz, the optimal *P*_local_ values is 2.175 mW, and $\delta x^{\mathrm{total}}$ has its smallest value of 4.2078 $\mathrm{pm}/\sqrt{\mathrm{Hz}}$.

In actual mission operations, a larger value of *P*_receive_ can not only reduce $\delta x^{\mathrm{total}}$, but also reduce the difficulty of weak-light phase-locked loops to some extent [27], which means that the power of *P*_tel_ must be increased. However, increasing *P*_tel_ undoubtedly increases the difficulty of the design of the devices associated with this system, and therefore, a trade-off needs to be made considering the practical applications of this system. In addition, different upper bounds of the beat-note frequency correspond to different optimal values of *P*_local_, and different minimum total readout displacement noise values. The value of *P*_local_ can be set by referring to Figure 6.

## 5. Conclusions

In this study, a hierarchical optimization algorithm is proposed to solve the Taiji program’s system-level frequency setting scheme. The optimization model considered the effects of six main factors, namely *f*_lower_, *f*_upper_, *f*_ADC_, *f*_PT_, *f*_USO_, and *m*. Two optimization objectives were used, including minimizing the total readout displacement noise and maximizing the feasible beat-note frequency range. Considering the characteristics involved in solving multi-objective optimization problems, 20 Pareto-optimal solutions were obtained. The minimum total readout displacement noise was 4.12 $\mathrm{pm}/\sqrt{\mathrm{Hz}}$, which corresponded to a beat-note frequency feasible range of 21 MHz, with (*f*_lower_, *f*_upper_, *f*_ADC_, *f*_PT_, *f*_USO_, *m*) = (3 MHz, 23 MHz, 96 MHz, 94 MHz, 4512 MHz, 0.44). The maximum feasible range of the beat-note frequency was 23 MHz with a total readout displacement noise of 4.21 $\mathrm{pm}/\sqrt{\mathrm{Hz}}$, with (*f*_lower_, *f*_upper_, *f*_ADC_, *f*_PT_, *f*_USO_, *m*) = (3 MHz, 25 MHz, 92 MHz, 90 MHz, 4140 MHz, 0.44). Hence, different values of the parameters *f*_upper_, *f*_ADC_, *f*_PT_, and *f*_USO_ result in different final optimization results. Therefore, these two objectives were not simultaneously optimized, and a trade-off between these two objectives needs to be made in practical applications of this system. Moreover, we analyzed the effects of *P*_tel_ and *f*_upper_ on *P*_receive_ and *P*_local_, and then explored the effects of these two factors on the total readout displacement noise. The results provide a reference for setting the frequency setting strategy during laser transmission and readout, determining the power ratio between the sidebands and carrier and selecting the relevant equipment parameters of laser interferometry systems in the Taiji program.

## Figures and Tables

**Table 1:** Taiji program parameters.

Parameter	Symbol	Value
Electron charge constant	*e*	1.6 × 10^−19^ C
Heterodyne interference efficiency	$\varepsilon_{\mathrm{het}}$	0.8 [20]
Photodiode responsivity	*R*_pd_	0.68 A/W [20]
Relative intensity laser noise	RIN	1 × 10^−8^ [21]
Photodetector phase number	*N*_pd_	4
Photodetector voltage noise	$\delta U_{\mathrm{pd}}$	$2\;\mathrm{nV}/\sqrt{\mathrm{Hz}}$
Photodiode capacitance	$C_{\mathrm{pd}}$	10 pF
Current noise	$\delta I_{\mathrm{pd}}$	$1.5\;\mathrm{pA}/\sqrt{\mathrm{Hz}}$ [22]
Laser power transmitted through the telescope	$P_{\mathrm{tel}}$	2 W [7]
Total optical efficiency	$\varepsilon_{\mathrm{opt}}$	0.853 [23]
Arm length	*L*	3 × 10^9^ m [6]
Diameter of telescope	*D*	40 cm [7]

**Table 2:** Time consumption of each step.

Steps	Algorithm	Time Consumption
Step 1	Nondominated sorting genetic algorithm II [24]	240 s
Step 2	Round	0.005 s
Step 3	Exhaustive enumeration	1 s
Step 4	Optimization	0.03 s

**Table 3:** Pareto-optimal feasible solutions obtained from hierarchical optimization algorithm.

Number	*f*_lower_ (MHz)	*f*_upper_ (MHz)	Feasible Range	*f*_ADC_ (MHz)	*f*_PT_ (MHz)	*f*_USO_ (MHz)	*m*	$\delta x^{\mathrm{total}}$ ($\mathrm{pm}/\sqrt{\mathrm{Hz}}$)	*P*_local_ (mW)
1	3	25	23	92	90	4140	0.44	4.21	2.06
2	3	25	23	63	61	3843	0.44	4.23	2.06
3	3	25	23	68	66	2244	0.48	4.47	2.06
4	3	25	23	62	60	1860	0.50	4.57	2.06
5	3	24	22	84	82	3444	0.44	4.24	1.99
6	4	25	22	71	68	4828	0.44	4.16	2.06
7	4	25	22	70	67	4690	0.44	4.17	2.06
8	3	24	22	65	63	4095	0.44	4.18	1.99
9	4	25	22	88	86	3784	0.44	4.24	2.06
10	4	25	22	59	57	3363	0.44	4.28	2.06
11	4	25	22	59	57	3363	0.44	4.28	2.06
12	4	25	22	70	68	2380	0.48	4.44	2.06
13	4	25	22	58	56	1624	0.52	4.65	2.06
14	3	23	21	96	94	4512	0.44	4.12	1.92
15	3	23	21	61	59	3599	0.44	4.19	1.92
16	4	24	21	65	62	4030	0.44	4.19	1.99
17	4	24	21	84	82	3444	0.44	4.24	1.99
18	4	24	21	88	86	3784	0.44	4.21	1.99
19	3	23	21	60	58	1740	0.50	4.52	1.92
20	5	25	21	59	57	3363	0.44	4.28	2.06

## Data Availability

Data underlying the results presented in this paper are not publicly available at this time but may be obtained from the authors upon reasonable request.
